# Mini review: Application of the somatic embryogenesis technique in conifer species

**DOI:** 10.48130/FR-2022-0018

**Published:** 2022-12-07

**Authors:** Tianqing Zhu, Junhui Wang, Jiwen Hu, Juanjuan Ling

**Affiliations:** 1 State Key Laboratory of Tree Genetics and Breeding, Key Laboratory of Tree Breeding and Cultivation of National Forestry and Grassland Administration, Chinese Academy of Forestry, Haidian District, Dongxiaofu 1, Beijing 100091, PR China; 2 State Key Laboratory of Tree Genetics and Breeding, Key Laboratory of Tree Breeding and Cultivation of National Forestry and Grassland Administration , Research Institute of Forestry, Chinese Academy of Forestry, Beijing 100091, PR China

**Keywords:** Conifer, Somatic embryogenesis, Molecular regulation, Large-scale propagation.

## Abstract

The somatic embryogenesis (SE) process is better suited to large-scale production and automation than other clonal propagation methods such as the rooting of cuttings. SE is becoming a key technique to promote the asexual industrialization of conifers. Furthermore, somatic embryos are an ideal material to study the molecular mechanism of conifer embryo development, as the processes of somatic and zygotic embryo development are very similar. This brief review introduces the culturing techniques of the SE process in conifers and outlines the progress and deficiencies in conifer SE research. Emphasis is placed on the patterning formation of conifer somatic embryos.

Somatic embryogenesis (SE) is a universal phenomenon unique to the plant kingdom. The SE technique has considerable application significance in valuable varieties for which it is difficult to obtain seeds and for forests with long growth cycles. The SE technique is considered to be one of the most important asexual propagation techniques for conifers^[1]^ and is conducive to the rapid reproduction of new varieties of valuable conifers. In 1985, Hakman et al. were the first to achieve SE in coniferous species^[2]^. They induced somatic proembryogenic masses (PEMs) using immature *Picea abies* zygotes as explants and obtained regenerated plants. To date, most conifer species can only use immature/mature embryos as explants for PEM induction, although there are a few exceptional genotypes in which PEM can be induced from primordial shoots^[3,4]^. The cell morphology and physiological changes associated with somatic embryo induction, maturation and germination of spruce species have been systematically studied in *Picea glauca*^[5]^. SE has been achieved in more than 50 tree species and hybrids in six coniferous genera, including *Abies, Larix, Picea, Pinus, Pseudotsuga* and *Sequol*^[2,5−8]^.

## Somatic embryo development in conifers

**Figure 1:**
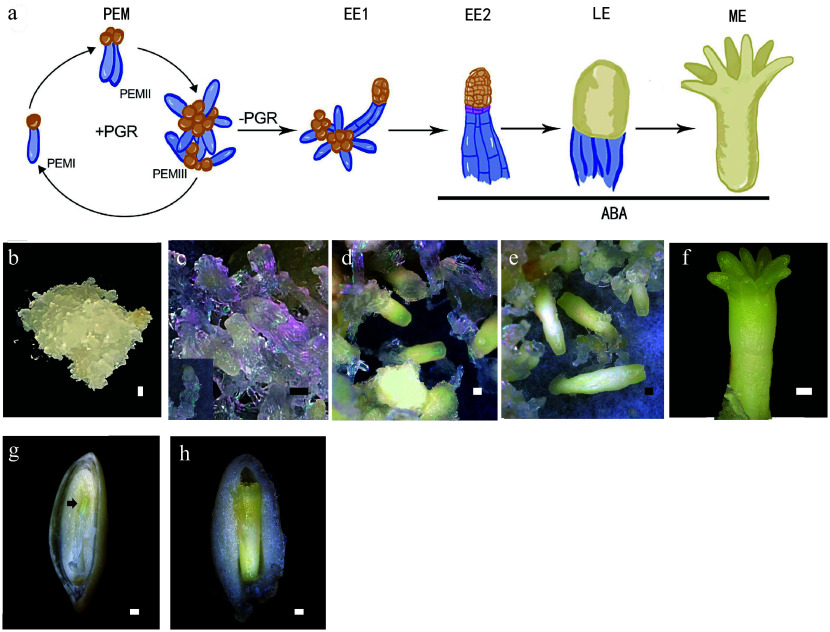
Conifer embryo development represented by *Picea abies*. (a) Schematic representation of the developmental stages of somatic embryo development. (b)−(f) Somatic embryo development process: (b) proembryogenic mass (PEM); (c) cultures after one week on maturation medium, insert presents an early somatic embryo (EE); (d) culture contains late embryos (LEs); (e) culture contains maturing and (f) matured somatic embryo (ME). (g), (h) Dissected seeds to show (g) the early zygotic embryo, indicated by the black arrow, and (h) the maturing zygotic embryo. Bar = 500 μm.

Conifer SE techniques involve four phases: proliferation of the PEM, induction of SE, formation of the meristematic centers, and development of the somatic embryo^[9]^. The PEM proliferates on a proliferation medium that contains plant growth factors (PGRs), including auxin and cytokinin. The cultures must be subcultured every 10 to 21 d (depending on the species) onto fresh medium. Long-term subculture leads to a decrease or even complete loss of embryo production capacity in the PEM lines. For long-term storage, the cultures can be cryopreserved. One week of withdrawal of PGRs effectively induces the differentiation of early somatic embryos (EEs). After the SE induction phase, the cultures are transferred to a maturation medium that contains abscisic acid (ABA) and a high sucrose content. The establishment of meristematic centers characterizes the formation of late somatic embryos (LEs). In the last phase, somatic embryos achieve both morphological and physiological maturity. It takes 4 to 6 weeks for development into mature somatic embryos (MEs) on maturation medium (Fig. 1a). The MEs must be desiccated before germination, and they are finally planted in the field.

## Induction of somatic embryogenesis in conifers

The induction and differentiation rates of the PEMs are related to genotype. Currently, some desirable varieties are difficult to propagate with the SE technique. Somatic dedifferentiation and redifferentiation require the regulation of a multitude of genes and chromosome reprogramming, which are controlled by DNA methylation and histone modification*.* Related research has been widely conducted in angiosperms. However, this knowledge is very limited in gymnosperms. Some genes have been verified as major regulators of SE or plant embryo patterning in a variety of plant species. Table 1 lists some of these genes that have been reported in conifers. Homologs of *BBM*, *LEC1*, *WOX2* and *SERK1* have been identified in *Larix decidua*^[10]^. *LEC1, WOX2* and *SERK1* are presumed to conserve their function in the induction of SE based on their expression pattern^[11]^.

**Table 1:** List of some of the major regulatory genes in somatic embryogenesis.

Gene family	Gene	Description	References
LRR-RLKs	*SOMATIC EMBRYOGENESIS RECEPTOR-* *LIKE KINASE 1-5 (SERKs)*	Transmembrane proteins; involved in signal transduction and have been strongly associated with somatic embryogenesis and apomixis in a number of plant species.	[23]
AP2/ERF	*BABYBOOM (BBM)*	Tanscription factor; activates LEC1-ABI3-FUS3-LEC2 network to induce somatic embryogenesis.	[24]
	*EMBRYOMAKER (EMK/AIL5 )*	Tanscription factors; promote the formation of somatic embryo on cotyledons.	[25]
	*WOUND INDUCED DEDIFFERENTIATION1 (WIND1)*	Tanscription factor; controls cell dedifferentiation in Arabidopsis and functions as a key molecular switch for plant cell dedifferentiation.	[26,27]
B3-AFL	*LEAFY COTYLEDON 1 (LEC1)*	Tanscription factor; promote somatic embryo development in vegetative organs.	[28]
	*LEC1-LIKE (L1L)*	Tanscription factor; promote somatic embryo development in vegetative organs.	[29]
	*LEAFY COTYLEDON 2 (LEC2)*	Tanscription factor; activates the expression of embryonic traits in vegetative tissues.	[28]
	*ABSCISIC ACID INSENSITIVE 3 (ABI3)/VIVI PAROUS (VP1)*	Transcript factor; regulates embryo-specific ABA-induced genes.	[30]
	*FUSCA3 (FUS3)*	Transcription factor; promotes embryogenesis by regulating synthesis of storage proteins and lipids.	[31]
	*VP1/ABI3-LIKE (VAL)*	Transcription factor; repress plant embryo development.	[32]
WOX	*WUSCHEL (WUS)*	Transcription factor; a central player in stem cell maintenance in the SAM.	[33]
	*WUSCHEL-related homeobox (WOX) 2*	Transcription factor; promotes apical embryonic cell division.	[34]
	*WOX 5*	Transcription factor; a central player in stem cell maintenance in the SAM.	[35]
	*WOX 8 and WOX9*	*WOX8* and *WOX9* functionally overlap in promoting basal embryonic cell division.	[34]
NAC	*CUP SHAPED COTYLEDONS 1-3 (CUCs)*	*CUP SHAPED COTYLEDONS 1-3* act redundantly to specify the cotyledon boundary.	[36−38]
HD-GL2	*Arabidopsis thaliana meristem L1 layer (ATML1)*	An early molecular marker for the establishment of both apical-basal and radial patterns during plant embryogenesis.	[39]
	*ANTHOCYANINLESS2 (ANL2)*	anl2 mutant shows aberrant cellular organization.	[40]
Class I KNOX gene	*SHOOTMERISTEMLESS (STM)*	Tanscription factors regulate the architecture of the SAM by maintaining a balance between cell division and differentiation.	[41]
GRAS	*SCARECROW (SCR)*	Regulates the radial organization of the root.	[42]
AGO proteins	ARGONAUTE (AGO)	Participate in post-transcriptional gene silencing and influence stem cell fate specification in both plants and animals.	[43]
PcG proteins	POLYCOMB REPRESSIVE COMPLEX subunit genes	Epigenetic effector proteins; stem cell self-renewal, pluripotency, gene silencing; repressive effect on dedifferentiation ability of cells.	[15,16]

Low levels of global DNA methylation have been found in the embryogenic cultures of several plants. It was found that *de novo* DNA methylation and its maintenance are required for the regulation of SE in *Picea abies*^[12]^. Klimaszewska et al. detected no significant differences in DNA methylation between embryogenic and nonembryogenic *Pinus pinaster* cultures^[13]^. Histone posttranslational modifications such as histone deacetylation and methylation have been implicated in the formation of somatic embryos. Trichostatin A (TSA) treatment, which inhibits histone deacetylases, interferes with somatic embryogenesis induction in conifers^[14]^. H3K27me3, which is written and read by polycomb repressive complex 2 (PRC2), controls cell differentiation by directing widespread transcriptional repression^[15,16]^. Nakamura et al.^[17]^ reported that the H3K27me3 level was low in the productive PEM but markedly increased upon embryo induction in *P. abies*.

Zygotic embryos are nourished *via* the phloem tissue, whereas somatic embryos use an exogenous supply of carbohydrates. It is assumed that the existence of ‘nurse cells’, which can provide an endosperm-like environment to facilitate the initial development of somatic embryos, is critical for the proliferation of PEMs. Conditioned medium (spent medium harvested from cultured cells) from embryogenic cultures can promote embryogenesis. Elhiti et al. reported 51 proteins that function in early somatic embryogenesis^[18]^. A glycosylated acidic endochitinase, which is involved in the cleavage of compounds such as lipo-chitooligosaccharides (LCOs)^[19]^ and arabinogalactan proteins (AGPs)^[20]^, can stimulate embryo development and growth. In *P. abies*, the chitinase 4-encoding gene Chia4-Pa is expressed in the single cell-layered zone surrounding the corrosion cavity of the megagametophyte and surrounding the early somatic embryo^[21]^. Furthermore, LCOs and AGPs have been isolated from *P. abies* conditioned medium and have been demonstrated to be effective stimulators of somatic embryogenesis^[19]^. Vanillyl benzyl ether has been confirmed to be an inhibitory compound that leads to the development of new somatic embryos^[22]^. This compound could inhibit the differentiation of suspensors.

## Comparison of zygotic and somatic embryo development in conifers

In angiosperms, asymmetric cell division of the zygote produces one cell that gives rise to the suspensor and another that give rise to the embryo proper. This phenomenon is not observed in SE. In conifers, zygotes undergo several rounds of nuclear duplication without cytokinesis to enter a free nuclear phase after fertilization, which is followed by cellularization to form an eight-celled proembryo. The cells of the apical portion multiply to form the embryo proper, whereas cells of the basal part elongate and undergo limited cell division to form the embryonic suspensor^[44]^. The free nuclear phase is absent in SE. The development of somatic embryos (Fig. 1b−f) is morphologically similar to that of zygotic embryos (Fig. 1g, h) in the later phases. After the induction of SE in the somatic embryo, the embryonal mass and the suspensor are separated by a layer of conifer-specific cells called embryonal tube cells. These cells produce apical meristem cells and basal suspensor cells through asymmetric anticlinal division. The primary body plan is established during embryogenesis. Somatic embryos are not hidden behind ovules and can be obtained throughout the year. These characteristics make somatic embryos an ideal material to study the physiological characteristics and molecular mechanism of conifer embryo development. Combining the relevant knowledge of angiosperms and reverse genetics, a general understanding of the molecular regulatory mechanism of conifer embryo development has been acquired.

## Patterning formation of conifer somatic embryos

Apical-basal differentiation is the foundation of plant embryonic development. Polar auxin transport is essential for the correct patterning of both the apical and basal parts of conifer embryos throughout the entire developmental process. Auxin transport inhibitor 1-N-naphthylphthalamic acid (NPA) treatment of early embryos leads to Indole-3-acetic acid (IAA) accumulation in the suspensor, which inhibits programmed cell death (PCD) of the suspensor, thereby resulting in aberrant suspensor development. NPA treatment of late embryos leads to fused cotyledons, the absence of shoot apical meristem (SAM) and aberrant root apical meristem (RAM)^[45]^. The aberrant morphologies of NPA-treated spruce embryos are comparable to several auxin response and transport mutants in Arabidopsis.

PCD eliminates unwanted cells during embryogenesis, which is necessary for correct embryonic pattern formation^[46]^. Two successive waves of PCD were observed during SE of *P. abies*^[47]^: the first wave was responsible for the degradation of ECs when they develop into somatic embryos; the second wave eliminated terminally differentiated embryo-suspensor cells during early embryogenesis. A reverse genetics study demonstrated that autophagic PCD is regulated by the type II metacaspase *mcII-Pa*. RNAi inhibition of *mcII-Pa* both inhibits autophagy in the suspensor cells and induces necrosis of the differentiated cells caused by autophagy disorder^[48,49]^.

WUSCHEL-related homeobox (WOXs) are a family of plant-specific transcription factors that play important roles in cell fate determination during plant development. There are 15 *WOX* genes in Arabidopsis. *AtWUS, AtWOX2, AtWOX5, AtWOX8* and *AtWOX9* are most relevant to embryo patterning. *AtWUS* and *AtWOX5* are crucial regulators of SAM and RAM, respectively^[35,50]^; they are necessary for meristem maintenance but are not required for their initiation. *AtWOX2* is specifically expressed in apical embryonic cells, whereas *AtWOX8* and *AtWOX9* are specifically expressed in suspensor cells after the first zygotic cell division^[34]^. Eleven and 14 *WOX* genes have been identified in *P. abies* and *P. pinaster,* respectively^[51,52]^. Notably, only one homolog of *WUS/WOX5* has been be detected in gymnosperms^[51,52]^. *P. pinaster WOX5* shows maximum expression at the mature embryo stage with transcripts preferentially located at the root tip of seedlings^[52]^. The *WUS* and *WOX5* genes are the result of angiosperm-specific gene duplication^[53]^. *PaWOX2* mRNA has been detected in the embryonal mass and upper suspensor during early embryogenesis. Functional studies show that *PaWOX2* conserves a function in protoderm formation during early embryo development and may exert a unique function in suspensor expansion in gymnosperms^[54]^. *PaWOX8/9*, a *P. abies* homolog of *AtWOX8* and *AtWOX9*, is highly expressed in PEMs and EEs. With the degradation of the suspensor, the expression level of PaWOX8/9 decreases gradually. Knockdown of *PaWOX8/9* by RNAi leads to aberrant cell division orientation in tube cells^[55]^. In addition, the transcript levels of some cell cycle-regulating genes such as *PaE2Fs* and *PaCYCBLs* are directly or indirectly regulated by *PaWOX8/9*. Cell cycle-regulating genes have a significant role in the regulation of asymmetric cell division^[56]^.

The outermost protoderm differentiates into the epidermis during embryogenesis^[57]^. Unlike angiosperms, conifer protoderm cells not only divide periclinally but also anticlinally. This makes it difficult to identify the epidermal layer of conifers. Several genes related to epidermal development in *P. abies* have been identified, including *PaWOX2*, *P. abies Homeobox1 (PaHB1)*, *PaHB2* and *Pa18,* a lipid transport protein (LTP) coding gene^[58]^. The expression patterns of *PaHB1* and *PaHB2* are similar to their Arabidopsis homologs, *AtML1* and *AtANL2*^[59]^. *AtML1* is a master regulator of epidermal cell fate. The expression of *AtML1* becomes restricted to the protoderm at the dermatogen stage^[39,60]^. Ectopic expression of *PaHB1* leads to early developmental arrest caused by a lack of protoderm^[59]^. *AtANL2* is involved in maintaining the subepidermal-layer identity^[40]^. *PaHB2* is uniformly expressed in PEMs and EEs. In MEs,* PaHB2* expression was mainly detected in the outermost layer of the cortex and the root cap^[59]^. However, it is still unclear if *PaHB2* is involved in the development of the cortex. LTPs are crucial for the formation of the cuticle layer^[61]^. *Pa18* is expressed in all cells in PEMs and its expression is restricted to the protoderm in developing embryos^[58]^.

The establishment of meristem centers are major patterning events during embryogenesis. STM, one of the four *KNOX1* family genes in Arabidopsis, is crucial for the establishment of the embryonal SAM^[41]^. Four KNOX1 genes, *HBK1*, *HBK2*, *HBK3* and *HBK4,* have been identified in *P. abies*^[62]^. *HBK2* and *HBK4* are expressed specifically in the SAM and are regulated by polar auxin transport^[63]^. *HBK1* and *HBK3* show more general expression patterns within the embryos^[64]^. Ectopic expression of the four *HBK* genes in transgenic Arabidopsis plants supports functions similar to those of *HBK2* and *HBK4* in SAM development^[63]^. Overexpression of *HBK3* in Arabidopsis leads to enlarged meristems and an expanded expression pattern of STM^[64]^. In addition, *HBK1* and *HBK3* are expressed in all tested PEM lines, whereas *HBK2* and *HBK4* are only expressed in cell lines that are competent to form mature embryos^[63]^*.* The* CUC* genes function in the formation of cotyledon boundaries and the establishment of the embryonal SAM^[36−38]^.* PaNAC01* and *PaNAC02* belong to the NAC gene family. Based on phylogenetic analysis, *PaNAC01* is more similar to *CUC1* and *CUC2* and can substitute for CUC2 in the Arabidopsis *cuc1cuc2* mutant^[65]^.

Several genes that are important regulators of root meristem in angiosperms have been shown to be expressed during somatic embryogenesis in conifers. *P. glauca AGO* (*PgAGO*) is expressed at the future site of RAM^[66]^. The highest expression level of conifer *AGO* homologs is detected at the late/mature transition stage of embryogenesis^[67]^. Furthermore, knockdown of *PgAGO* leads to abnormal root meristems. The expression patterns of SCR, SHORT-ROOT (SHR) and several SCR-likes (SCLs) show expression patterns similar to their Arabidopsis homologs in several conifer species. SCR from the GRAS transcription factor family is important for the early delineation of radial patterning in the embryonic root^[68]^.

A genomics study based on conifer expressed sequence tag (EST) collections shows that the conifer embryo differs markedly from other gymnosperm tissues studied in terms of the range of genes transcribed. Approximately 72% of conifer embryo-expressed genes are found in Arabidopsis and have sequences similar to key genes that regulate seed development in Arabidopsis. However, approximately 11% of *Pinus taeda* embryo ESTs are novel^[44]^. The first conifer genome, the *P. abies* genome, was published in 2013^[69]^. The genomes of other conifers such as *P. glauca*^[70]^ and *Pinus tabuliformis*^[71]^ have been subsequently published. The sizes of the conifer genomes range from approximately 6,500 to 37,000 Mb and are highly repetitive. These factors make the genomes difficult to fully assemble. With the improvement of the genome and other biological information, a better understanding of the molecular mechanism of embryogenesis in conifers will definitely benefit the development of SE techniques.

## Conifer somatic embryogenesis techniques and the forest industry

The SE technique could intervene at two stages of the forest breeding strategy. First, the SE technique could be used to achieve faster offspring determination by providing an accurate assessment of genotype stability. Second, after the candidate genotype is identified, the SE technique could be used to mass produce valuable genotype copies and eventually achieve large-scale production. Forestry breeding *via* SE has several advantages compared with traditional forest breeding: 1) SE is not affected by the flowering and seed production cycle of forest trees, which provides greater flexibility for the deployment of forest renewable resources; 2) the intensity of genetic selection is greatly improved by SE breeding, as it is possible to achieve rapid reproduction of a small number of genotypes, which have a larger selection differential; 3) the SE technique combined with early selection on molecular labels could be used in the early development stage to better evaluate phenotypic type and plasticity and eventually shorten the breeding cycle; and 4) evaluating the traits of SE seedlings provides stronger evidence for genetic assays than traditional progeny tests. Using SE seedling evaluation, the environmental interactions with genotypes can be estimated more accurately, which improves the efficiency of clone determinations^[6]^.

Information on the application of the SE technique in industrial production is still limited at present. Tree species such as *Abies nordmanniana, P. abies, P. glauca, P. sitchensis, Pseudotsuga menziesii, Pinus radiata* and* P. taeda* are being researched. *P. abies,* *P. sitchensis,* *P. menziesii, P. radiata* and* P. taeda* are of interest for the commercial production of coniferous SE plants by transnational corporations. According to reports, the Arborgen (USA) and Weyerhaeuser (USA) companies, which are among the world's largest wood producers, have the largest application capacity for the SE technique. Arborgen could produce one million *P. taeda* SE seedlings annually. Weyerhaeuser plans to produce ten million synthetic seeds per year *via* the SE technique.

It is still a long process to achieve industrialization of conifer somatic embryo production. Establishing a cryopreserved PEM library would be conducive to applying the SE technique to industrial production. In addition, plant biologists strive to expand the explant types that can be used for PEM induction. Finally, determining how to reduce costs in the SE process without affecting the quality of embryos is a problem that concerns many companies and institutions. In addition to improving the methods of PEM induction, somatic embryo differentiation, germination and planting, it is also important to connect each step effectively to maximize the production efficiency while minimizing the economic cost. Several strategies have been investigated including strict control of liquid proliferative media conditions and embryonic tissue density for different genotypes to maintain consistency in callus growth and proliferation cycle duration and manual control of the environmental conditions of germination to reduce the germination time *in vitro* without affecting the germination percentage. Egertsdotter et al. have summarized these studies and the progress of the SE technique in the field of conifer breeding^[72]^. Establishing a database and developing an automated system to monitor the status of cryopreservation and production materials, mechanizing the production process of SE, applying bioreactors and automation systems in the production of somatic embryos and studying the impact of light on the development of somatic seedlings would also be essential tasks to achieve the breeding goal of using the SE technique to achieve efficient, high-quality and economical production of tens of millions of seedlings.
